# Comparative effects of recovery strategies on exercise-induced muscle fatigue:a randomized controlled trial

**DOI:** 10.3389/fphys.2025.1622669

**Published:** 2025-10-10

**Authors:** Ming Wei, Xudong Shen, Siyu Wang

**Affiliations:** Shenyang Sport University, Shenyang, China

**Keywords:** exercise-induced muscle fatigue, recovery and regeneration, massage, vibration therapy, cold-water immersion

## Abstract

**Objective:**

This study aimed to compare the effects of five post-exercise recovery interventions—massage, cold-water immersion (CWI), vibration therapy, functional electrical stimulation (FES), and static stretching—on central nervous modulation, neuromuscular function recovery, and inflammatory response following exercise-induced muscle fatigue (EIMF).

**Methods:**

This randomized controlled trial employed a two-way repeated-measures analysis of variance (ANOVA; group × time) to evaluate the effects of different recovery interventions over time. Thirty healthy male participants were randomly assigned to six groups (n = 5 each): massage (Group A), CWI (Group B), vibration therapy (Group C), static stretching (Group D), FES (Group E), and control (Group F). EIMF was induced using a standardized exercise model. Physiological and biochemical measurements were taken at baseline, immediately post-exercise, and at 24, 48, and 72 h. Physiological indicators included muscle contraction time (TC), maximal radial displacement (DM),peak concentric power, The blood biochemical indicators include gamma-aminobutyric acid (GABA), creatine kinase (CK),and interleukin-6 (IL-6).

**Results:**

Vibration therapy demonstrated a significant advantage in reducing TC at 72 h post-exercise (p = 0.027,p < 0.05); Although no statistically significant differences were found for DM,the massage group showed a more favorable recovery trend; FES significantly improved peak concentric power at 48 h post-exercise (p = 0.000, p < 0.01). Massage significantly increased GABA levels (p = 0.001, p < 0.05) and reduced CK concentrations (p = 0.000, p < 0.01) at 48 h. CWI demonstrated a significant inhibitory effect on IL-6 at 48 h (p = 0.000, p < 0.01).

**Conclusion:**

Massage therapy showed favorable effects in central modulation and muscle repair. CWI effectively controlled acute inflammation. FES improved muscle explosive power, while vibration therapy enhanced neuromuscular responsiveness. Static stretching group showed no significant recovery benefit, as stretching primarily enhances ROM and flexibility and has only limited impact on the critical physiological pathways necessary for EIMF recovery.

## 1 Introduction

EIMF refers to a transient decline in the ability of muscles to generate maximal contractile force or power output (Pedersen and Febbraio, 2008). It results from a combined reduction in peripheral contractile function and central neuromuscular activation capacity (Nybo and Secher, 2004). Studies have shown that brain fatigue is a major contributor to central nervous system dysfunction (Zając et al., 2015), with changes in serotonin (5-HT) (Meeusen et al., 2006), dopamine, glutamate (Glu), and GABA serving as key biochemical markers for monitoring central regulation (Tomporowski, 2003). Peripheral fatigue, in contrast, is primarily induced by biochemical alterations during muscular activity—including changes in inorganic phosphate, calcium ions, lactate, ADP, and magnesium—which diminish neuromuscular transmission efficiency (Ament et al., 2009). The accumulation of these metabolites may suppress myofibrillar ATPase activity, disrupt actin–myosin cross-bridge cycling, and impair calcium reuptake by the sarcoplasmic reticulum, ultimately resulting in reduced muscle force output.

Although EIMF typically resolves within a few days, factors prevalent in competitive sports (Zając et al., 2015)—such as dense competition schedules (Wiewelhove et al., 2022), high training loads (Mclaren et al., 2018), and inadequate recovery (Carroll et al., 1985)—can cause this fatigue to transition into musculoskeletal injuries, consequently impairing athletic performance (Halson, 2014; Spencer et al., 2005; Abd-Elfattah et al., 2015). Therefore, the timeline for recovery is a critical factor, and the application of timely and targeted interventions is essential for accelerating physiological regeneration. Various recovery interventions have been widely implemented to alleviate EIMF, including massage (Weerapong et al., 2005; Poppendieck et al., 2016), cryotherapy (Wang et al., 2025), FES (Babault et al., 2011; Piras et al., 2021), static stretching (Afonso et al., 2021), and vibration therapy (Bakhtiary et al., 2007). Among these strategies, massage alleviates fatigue by increasing blood flow (Bakar et al., 2015), reducing muscle edema, lowering cortisol levels, and elevating dopamine and β-endorphin concentrations through specific manual techniques (Field et al., 2005). CWI stimulates the peripheral parasympathetic nervous system, induces vasoconstriction, enhances muscle blood flow, improves pulmonary ventilation, and increases oxygen uptake, thereby promoting recovery after high-intensity exercise (Abaïdia et al., 2017; Leppäluoto et al., 2008). FES mimics neural signals by modulating pulse type and current intensity to induce rhythmic muscle contraction and relaxation, thereby promoting muscle relaxation and recovery (Esteve et al., 2017). Static stretching reduces EIMF by altering neuromuscular activation and modulating the tension in muscles and tendons. Finally, vibration therapy (Bakhtiary et al., 2007), through specific frequencies and durations, can modulate the sensitivity of the stretch reflex and influence the excitation threshold of motor units to regulate muscle tone and force output.

This study only included healthy male college students to minimize the influence of sex-related variables. Previous studies have shown significant differences between males and females in neuromuscular performance and fatigue capacity, with females also being influenced by menstrual hormones, which could introduce substantial variability in experimental outcomes (Mcnulty et al., 2020). Therefore, limiting the participants to males was more conducive to evaluating the effects of the different interventions.

Although these modalities have shown some effectiveness in mitigating muscle fatigue, the specific effects and mechanisms by which they influence central and peripheral fatigue remain unclear. This knowledge gap hinders the evidence-based selection or combination of recovery strategies for optimizing post-exercise regeneration.

## 2 Materials and methods

### 2.1 Study design

**FIGURE 1:**
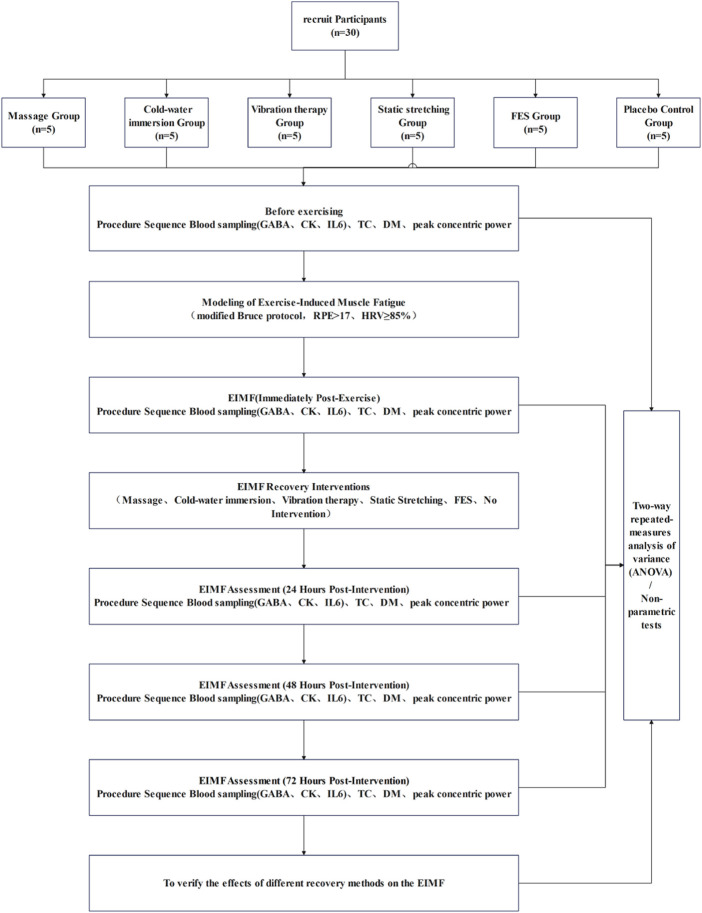
Study workflow.

This randomized, single-blind (assessor-blinded), controlled trial was conducted at the Strength and Conditioning Center of Shenyang Sport University. Because the recovery modalities were perceptible to participants (e.g., CWI, massage, vibration, FES, and static stretching), blinding of participants and interventionists was not feasible. To minimize risk of bias, outcome assessors and laboratory technicians were blinded under de-identification procedures. Specifically, the intervention and assessment teams were separated; interventions and tests were delivered according to standardized protocols; all test results were labeled with coded identifiers; and statisticians remained blinded until database lock, analyzing de-identified group codes (Figure 1). Throughout the study, all procedures followed standardized operating procedures, and no adverse events occurred.

### 2.2 Participants

**FIGURE 2:**
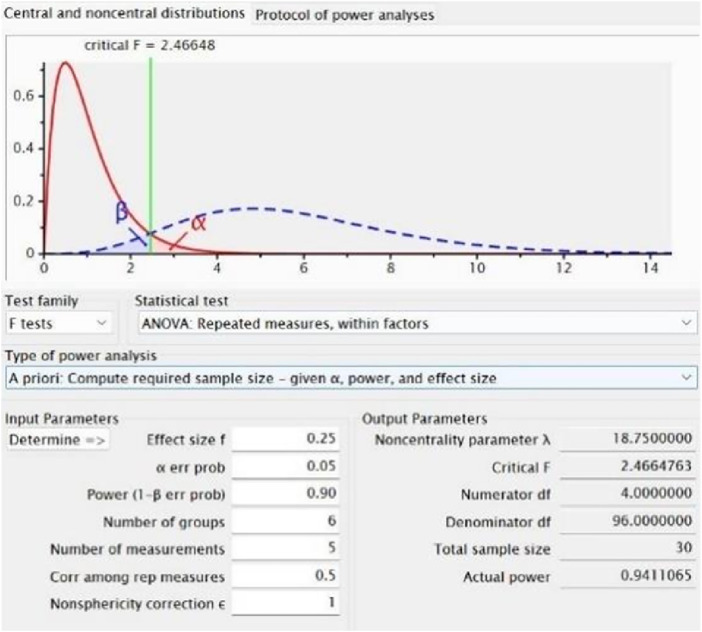
G*Power 3.1 output for sample size estimation based on repeated-measures ANOVA.

*A priori* sample size estimation was performed using G*Power 3.1, based on pilot data and conservative estimates derived from existing literature. An effect size of 0.25 and a statistical power of 0.90 were adopted for the calculation (Figure 2). The test family was set to “F tests” and the statistical test selected was “ANOVA: Repeated measures, within factors.” Parameters were specified as follows: effect size f = 0.25, α = 0.05, power (1−β) = 0.90, number of groups = 6, number of measurements = 5, correlation among repeated measures = 0.5, and nonsphericity correction ε = 1. Based on the calculation, a total of 30 participants were required。Randomization was performed using a computer-generated sequence without prior allocation, and participants were assigned to six groups: massage, CWI, FES, static stretching, vibration therapy, and control (n = 5 per group).

Thirty healthy male university and graduate students (aged 18–28 years) from Shenyang Sport University were recruited Participants were required to abstain from heavy training, competition, alcohol consumption, and sleep deprivation for at least 48 h prior to testing. A general medical screening was conducted to exclude any clinical conditions or injuries that would contraindicate high-intensity physical activity. All participants were fully informed of the experimental procedures and risks, familiarized with the exercise protocol, and provided written informed consent prior to participation. Baseline characteristics of the participants are shown in Table 1, with no significant differences observed among the six groups.

**TABLE 1:** Baseline characteristics of participants across six groups (mean ± SD).

Group	n	Age (years)	Height (cm)	Weight (kg)
A	5	21.80 ± 1.30	177.60 ± 4.04	78.00 ± 4.69
B	5	22.60 ± 1.34	178.20 ± 3.96	77.00 ± 6.04
C	5	22.20 ± 1.92	181.60 ± 4.98	75.40 ± 7.83
D	5	22.00 ± 1.58	176.60 ± 2.70	72.00 ± 4.64
E	5	23.00 ± 1.87	179.20 ± 4.76	76.60 ± 5.59
F	5	22.00 ± 1.58	177.60 ± 4.39	71.80 ± 6.98
Total	30	22.26 ± 1.53	177.60 ± 4.04	78.00 ± 4.69

Data are presented as mean ± SD., No significant differences between groups (one-way ANOVA): Age: P = 0.853, Height:P = 0.515, Weight: P = 0.470.

### 2.3 Exercise-induced muscle fatigue protocol

All participants completed a 10-min warm-up, consisting of three sets of 30 s of foam rolling exercises and 5 min of dynamic stretching. EIMF was induced using a modified Bruce protocol (Mcinnis and Balady, 1994; Miller et al., 2007; Fletcher et al., 2001; Ratter et al., 2014), implemented on a Woodway treadmill (United States of America). The protocol began at a speed of 2.7 km/h with a 0% incline and increased in grade and speed every 3 min across 9 levels, reaching a maximum speed of 9.6 km/h and an incline of 22%.Total exercise duration is 28–34 min.

The modified Bruce protocol is designed to push an individual’s oxygen uptake towards its maximum (VO_2_ max), inducing significant stress on the cardiovascular, respiratory, and metabolic systems. This not only leads to peripheral muscle fatigue due to the accumulation of metabolic byproducts but also generates physiological stress, triggering central nervous system fatigue. The protocol has been validated in previous studies and shown to reliably induce muscle fatigue under controlled conditions.

### 2.4 Fatigue criteria

During the protocol, immediate heart rate was recorded using the Polar heart rate telemetry system (Finland), and subjective fatigue was assessed using the Rating of Perceived Exertion (RPE) scale. EIMF was confirmed when either of the following criteria were met: (1) Heart rate exceeded 85% of the predicted maximum (220 − age) (Costa et al., 2022); (2) RPE score reached 17 or higher (Arney et al., 2019). In addition, a significant reduction in peak concentric power, along with observable changes in TC and DM immediately after exercise, were considered supplementary evidence supporting the successful induction of EIMF.

### 2.5 Recovery interventions

The specific procedures for each intervention are detailed in Table 2. To ensure that each intervention was applied in the most effective manner, the duration, frequency, and intensity of each intervention were determined based on existing high-quality research and clinical practice guidelines in the field.

**TABLE 2:** Dosage parameters of recovery interventions.

Intervention	Device/Type	Stimulation parameters	Duration per session
massage (Guo et al., 2017)	Deep longitudinal pressure	Pressure: 250–300 kPa Frequency: 10strokes/min Pain control:VAS 5-6/10	10 min
CWI (Xiao et al., 2023)	Water bucket	Temperature:11 °C–15 °C; Immersion depth: up to iliac crest	12 min
Vibration therapy (Ferreira et al., 2023)	DMS	Frequency:60 Hz	10 min
Static stretching (Bryant et al., 2023)	Passive static stretch	Intensity: 80% of ROM; Set interval: 30s	30s/per muscle group×3sets
FES (Nussbaum et al., 2017)	Compex® Sport Elite5.0	Pulse width:250 μs; Current:30–45mA; Duty cycle:5s on/10s off	20 min

Massage: Manual therapy was applied to the quadriceps for 10 min, adjusting pressure to a tolerable range (VAS 5–6).

CWI: Participants were submerged in 11 °C–15 °C water for 11–15 min.

FES: A Compex Sport Elite5.0 device was used in “Relaxing Massage” mode on gluteal, quadriceps, hamstrings, tibialis anterior, and triceps surae muscles. Intensity was auto-adjusted based on muscle status; total duration was approximately 22 min.

Static Stretching: Targeted static stretching was performed for the gluteals, quadriceps, hamstrings, tibialis anterior, and triceps surae. Each stretch was held for 1 min with controlled breathing.

Vibration Therapy: A deep muscle stimulator (DMS) was applied bilaterally to the gluteal muscles, quadriceps, hamstrings, tibialis anterior, and triceps surae at a frequency of 60 Hz. Each muscle group received vibration treatment for 3 min.

Control Group: No intervention was administered.

### 2.6 Outcome measures

In accordance with CONSORT reporting guidelines, outcomes were prespecified as primary or secondary based on their relevance to the study hypothesis. All outcomes were assessed at five time points: baseline, immediately post-exercise, and 24, 48, and 72 h post-intervention. The primary outcome was quadriceps peak concentric power, which directly reflects recovery of muscle function following exercise-induced muscle fatigue and served to determine the primary efficacy of each recovery intervention. For isokinetic testing, participants were seated on an isokinetic dynamometer (ISOMed 2000, Germany) with the greater trochanter aligned to the axis of rotation; the trunk, pelvis, and distal third of the thigh were firmly stabilized, and participants grasped the side handles. Testing was performed at an angular velocity of 240°·s^-1^ and consisted of five maximal concentric contractions; the highest value was retained for analysis.

Secondary outcomes comprised physiological and biochemical markers. The physiological endpoints were TC and DM, assessed by TMG to evaluate neuromuscular responsiveness and muscle stiffness. All TMG measurements were performed by the same experienced technician under a strict standardized protocol; the rectus femoris was stimulated with a single electrical pulse (duration 1 m; intensity 20–80 mA). The biochemical endpoints were GABA, CK, and IL-6, reflecting central neural modulation, muscle damage, and inflammatory responses, respectively. Venous blood (3 mL) was drawn from the antecubital vein by a trained nurse, centrifuged at 3,000 rpm for 15 min using a benchtop centrifuge (TGL-16c, Shanghai, China) to separate serum, immediately aliquoted, and stored in liquid nitrogen at −196 °C until analysis. Serum concentrations were quantified with a fully automated biochemical analyzer according to standardized protocols.

### 2.7 Statistical analysis

All statistical analyses were performed using SPSS version 27.0 (IBM Corp, Armonk, NY, United States). Data were presented as mean ± standard deviation (SD). A two-way repeated-measures analysis of variance (ANOVA) was conducted to evaluate the effects of different recovery interventions and time points on the following outcome variables: TC, DM,peak concentric power, GABA,CK, and IL-6.

Baseline comparisons were conducted to assess group comparability after randomization. Demographic and clinical characteristics were compared across groups. The Shapiro–Wilk test was used to assess the normality of continuous variables. Normally distributed variables were analyzed using parametric tests, while non-normally distributed variables were analyzed using the Mann–Whitney U test. Categorical variables were compared using the chi-square test. When the expected frequency of any cell was less than 20% of the total sample, Fisher’s exact test was applied. All data were expressed as mean ± standard deviation (SD), and statistical significance was set at p < 0.05. Baseline characteristics that showed significant between-group differences were used as covariates in the main efficacy and safety analyses after randomization.

When significant interaction effects were detected, simple effects analyses were performed to further investigate the main effects. The Bonferroni *post hoc* test was applied to compare differences between groups and across time points for all assessed variables. The significance level was adopted at α = 0.05 for all statistical analyses.

## 3 Results

### 3.1 Physiological assessments

#### 3.1.1 TC

Superscript annotations:*a*: *p* < 0.05 vs. control group at the same time point;*aa*: *p* < 0.01 vs. control group at the same time point;*b*: *p* < 0.05 vs. pre-exercise within the same group;*bb*: *p* < 0.01 vs. pre-exercise within the same group;*c*: *p* < 0.05 vs. immediate post-exercise within the same group;*cc*: *p* < 0.01 vs. immediate post-exercise within the same group;*d*: *p* < 0.05 vs. 24 h post-exercise within the same group;*dd*: *p* < 0.01 vs. 24 h post-exercise within the same group.

Shapiro–Wilk tests confirmed that the TC data in all groups followed a normal distribution. Results from the two-way repeated-measures ANOVA are presented in Table 3. The interaction effect between time and group was not significant (p > 0.05, partial η^2^ = 0.257). A significant main effect of time was observed (p < 0.01, partial η^2^ = 0.479), whereas the main effect of group was not significant (p > 0.05, partial η^2^ = 0.101).

**TABLE 3:** Changes in TC Across Groups and Time Points (mean ± SD, ms).

Group	Pre-exercise	Post-instant	Post-24 h	Post-48 h	Post-72 h	Time effect: F	Group effect: F	Interaction effect: F
A	21.82 ± 2.53	29.05 ± 5.08^b^	25.11 ± 6.27	21.65 ± 2.30^c^	21.77 ± 2.34^c^	22.061	0.541	1.657
B	21.72 ± 1.65	26.8 ± 3.50^bb^	24.10 ± 4.08	23.50 ± 4.21	22.46 ± 3.06			
C	21.36 ± 2.58	30.38 ± 2.55^b^	23.27 ± 2.44^cc^	21.97 ± 2.39^cc^	19.35 ± 0.86^acc^			
D	20.86 ± 4.84	26.96 ± 5.22^b^	26.61 ± 5.21	25.16 ± 2.56	24.51 ± 1.13			
E	23.85 ± 3.21	28.01 ± 1.45^b^	23.73 ± 5.52	22.94 ± 4.94^c^	24.31 ± 4.99			
F	23.54 ± 2.43	28.12 ± 2.06	24.16 ± 3.65	26.61 ± 2.70	25.62 ± 2.41			
P						0.000	0.743	0.076

A = massage group; B = CWI, group; C = vibration therapy group; D = static stretching group; E = FES, group; F = Control group. Same group abbreviations apply in subsequent tables.

Although no statistically significant main effect of group was observed, analysis at 72 h post-exercise revealed that the vibration therapy group had significantly lower TC values compared to the control group (p = 0.027, p < 0.05, 95% CI:−8.91, −3.63). Descriptive statistics also indicated that the vibration group showed the lowest TC values among all groups, suggesting a potential advantage in reducing peripheral fatigue.

#### 3.1.2 DM

Shapiro–Wilk tests confirmed that the DM data in all groups followed a normal distribution. The results of the two-way repeated-measures ANOVA are shown in Table 4. A significant interaction effect between time and group was found (p < 0.05, partial η^2^ = 0.371), along with a significant main effect of time (p < 0.01, partial η^2^ = 0.859). The main effect of group was not statistically significant (p > 0.05, partial η^2^ = 0.165).

**TABLE 4:** Changes in DM Across Groups and Time Points (mean ± SD, mm).

Group	Pre-exercise	Post-instant	Post-24 h	Post-48 h	Post-72 h	Time effect:F	Group effect:F	Interaction effect:F
A	7.38±0.45	2.49±0.74^bb^	5.38±0.76^b^ ^,^ ^cc^	6.25±0.41^cc^	6.59±0.57^cc^	146.117	0.950	2.828
B	7.59±0.81	2.78±1.20^bb^	3.20±1.08^bb^	3.98±0.41^bb^	4.42±1.00^bb^ ^,^ ^c^			
C	8.56±0.87	3.28±0.80^bb^	4.64±1.60^bb^ ^,^ ^c^	4.46±0.78^bb^	5.54±0.55^bb^ ^,^ ^cc^			
D	7.76±1.50	3.0±2.02^bb^	3.39±2.55^bb^	4.26±2.90^bb^	4.93±2.80^bb^ ^,^ ^c^			
E	8.30±0.57	4.31±2.01^bb^	4.45±1.04^bb^	4.38±0.91^bb^	5.79±1.82^bb^ ^,^ ^c^			
F	8.30±0.98	2.93±0.76^bb^	3.34±1.33^bb^	4.17±1.48^bb^	4.38±1.38^bb^ ^,^ ^c^			
P						0.000	0.468	0.001

Superscript annotations ^a^: *p* < 0.05 vs. control group at the same time point.

aa : *p* < 0.01 vs. control group at the same time point.

b : *p* < 0.05 vs. pre-exercise within the same group.

bb : *p* < 0.01 vs. pre-exercise within the same group.

c : *p* < 0.05 vs. immediate post-exercise within the same group.

cc : *p* < 0.01 vs. immediate post-exercise within the same group.

d : *p* < 0.05 vs. 24h post-exercise within the same group.

dd : *p* < 0.01 vs. 24h post-exercise within the same group.

Although no significant group differences were observed overall, we noted variations in DM recovery across different time points. Descriptive statistics indicated that, compared to the other groups, the massage group’s average DM value at 48 h post-exercise was closest to baseline levels.

#### 3.1.3 Peak concentric power results

Shapiro–Wilk tests confirmed that the peak concentric power data in all groups followed a normal distribution. Results from the two-way repeated-measures ANOVA are summarized in Table 5. A significant interaction effect between time and group was observed (p < 0.05, partial η^2^= 0.340), as well as a significant main effect of time (p < 0.01, partial η^2^= 0.800) and a significant main effect of group (p < 0.05, partial η^2^= 0.468).

**TABLE 5:** Changes in peak concentric power across groups and time points (mean ± SD, W/ms).

Group	Pre-exercise	Post-instant	Post-24 h	Post-48 h	Post-72 h	Time effect:F	Group effect:F	Interaction effect:F
A	196.68±10.93	132.42±20.89^b^	161.86±18.29^bb^ ^,^ ^c^	170.72±15.00^a^ ^,^ ^bb^ ^,^ ^c^	183.03±10.46^bb^ ^,^ ^cc^ ^,^ ^d^	95.948	4.255	2.473
B	183.87± 8.54	131.71±19.86^b^	161.85±6.01^b^ ^,c^	167.05±8.75^a^ ^,^ ^b^ ^,^ ^c^	173.92±16.45^c^			
C	187.71± 9.42	133.60±18.33^bb^	162.03±15.46^b^ ^,^ ^c^	169.09±7.68^a^ ^,^ ^b^ ^,^ ^c^	169.88±18.15^c^			
D	188.23±11.66	131.51±22.05^bb^	166.17±7.67^a^ ^,^ ^b^ ^,^ ^c^	170.60±10.10^a^ ^,^ ^b^ ^,^ ^c^	163.43±14.97^b^ ^,^ ^c^			
E	197.85±12.03	139.65±23.30^b^	189.09±12.16^a^ ^,^ ^cc^	191.81±11.24^a^ ^,^ ^cc^	197.45±21.60^a^ ^,^ ^cc^			
F	192.97± 9.23	119.50±36.84^bb^	130.35±27.56^bb^	134.94±20.01^bb^	153.46±19.49^bb^ ^,^ ^c^ ^,^ ^d^			
P						0.000	0.007	0.009

Shapiro–Wilk tests confirmed that the peak concentric power data in all groups followed a normal distribution. Results from the two-way repeated-measures ANOVA are summarized in Table 5. A significant interaction effect between time and group was observed (p < 0.05, partial η^2^ = 0.340), as well as a significant main effect of time (p < 0.01, partial η^2^ = 0.800) and a significant main effect of group (p < 0.05, partial η^2^ = 0.468).

Superscript annotations ^a^: *p* < 0.05 vs. control group at the same time point.

aa : *p* < 0.01 vs. control group at the same time point.

b : *p* < 0.05 vs. pre-exercise within the same group.

bb : *p* < 0.01 vs. pre-exercise within the same group.

c : *p* < 0.05 vs. immediate post-exercise within the same group.

cc : *p* < 0.01 vs. immediate post-exercise within the same group.

d : *p* < 0.05 vs. 24h post-exercise within the same group.

dd : *p* < 0.01 vs. 24h post-exercise within the same group.

Post hoc multiple comparisons revealed that the FES group exhibited the most effective recovery in terms of restoring peak concentric power following exercise-induced muscle fatigue, with a statistically significant improvement observed at 48 h post-exercise (p = 0.000, p < 0.01, 95%CI:33.20, 80.54).

### 3.2 Blood biochemical analysis

#### 3.2.1 GABA

Shapiro–Wilk tests confirmed that the GABA data for all groups followed a normal distribution. Results from the two-way repeated-measures ANOVA are shown in Table 6. The interaction effect between time and group was not significant (p > 0.05, partial η^2^ = 0.244). However, there was a significant main effect of time (p < 0.01, partial η^2^ = 0.838), whereas the main effect of group did not reach statistical significance (p > 0.05, partial η^2^ = 0.446).

**TABLE 6:** Changes in GABA Concentration Across Groups and Time Points (mean ± SD, pg/mL).

Group	Pre-exercise	Post-instant	Post-24 h	Post-48 h	Post-72 h	Time effect: F	Group effect: F	Interaction effect: F
A	4.47±1.25	11.20±2.06^b^	7.69±0.90^b^	3.34±0.92^a^ ^,^ ^c^ ^,^ ^d^	4.67±0.62^c^ ^,^ ^d^	124.117	3.869	1.553
B	4.62±1.04	14.84±3.28^bb^	7.55±1.45^b^ ^,^ ^c^	5.03±1.06^a^ ^,^ ^cc^	4.51±1.40^cc^ ^,^ ^d^			
C	3.57±0.88	14.45±3.07^bb^	9.62±2.43^bb^	4.88±1.13^a^ ^,^ ^cc^	5.35±0.88^cc^			
D	4.24±0.78	13.40±2.21^bb^	10.12±1.68^bb^	5.76±1.32^c^ ^,^ ^d^	4.77±1.06^cc^ ^,^ ^d^			
E	3.99±0.71	13.66±3.20^bb^	9.63±1.10^bb^	5.93±1.65^c^ ^,^ ^d^	4.84±1.05^cc^ ^,^ ^dd^			
F	3.65±1.11	12.75±3.44^bb^	10.53±2.73^bb^	8.44±2.85^bb^	5.66±1.78^b^ ^,^ ^c^ ^,^ ^dd^			
P						0.000	0.10	0.139

Superscript annotations ^a^: *p* < 0.05 vs. control group at the same time point.

aa : *p* < 0.01 vs. control group at the same time point.

b : *p* < 0.05 vs. pre-exercise within the same group.

bb : *p* < 0.01 vs. pre-exercise within the same group.

c : *p* < 0.05 vs. immediate post-exercise within the same group.

cc : *p* < 0.01 vs. immediate post-exercise within the same group.

d : *p* < 0.05 vs. 24h post-exercise within the same group.

dd : *p* < 0.01 vs. 24h post-exercise within the same group.

Although neither the main effect of group nor the interaction effect reached statistical significance, analysis at 48 h post-exercise revealed that the massage group had significantly lower GABA levels compared to the control groups (p = 0.001, p < 0.05, 95%CI:−8.19,−2.01). Descriptive statistics further indicated that the GABA level in the massage group was the lowest among all interventions, suggesting a more favorable trend in alleviating central fatigue.

#### 3.2.2 IL-6

Shapiro–Wilk tests confirmed that the IL-6 data across all groups followed a normal distribution. The results of the two-way repeated-measures ANOVA are presented in Table 7. A significant interaction effect between time and group was observed (p < 0.01, partial η^2^= 0.472), along with significant main effects of both time (p < 0.01, partial η^2^= 0.854) and group (p < 0.01, partial η^2^= 0.733).

**TABLE 7:** Changes in IL-6 Concentration Across Groups and Time Points (mean ± SD, pg/mL).

Group	Pre-exercise	Post-instant	Post-24 h	Post-48 h	Post-72 h	Time effect:F	Group effect:F	Interaction effect:F
A	165.53± 17.21	272.81± 28.70^bb^	229.09± 29.30^a^ ^,^ ^b^	187.01±1 3.12^a^ ^,^ ^c^	169.15± 9.02^aa^ ^,^ ^cc^	140.088	13.167	4.298
B	166.75± 13.06	284.56± 20.26^bb^	184.70± 31.42^aa^ ^,^ ^cc^	148.85± 22.13^aa^ ^,^ ^cc^	166.06± 7.31^aa^ ^,^ ^cc^			
C	172.54± 19.13	285.58± 40.81^bb^	239.87± 34.78^a^ ^,^ ^b^ ^,^ ^c^	198.56± 10.63^c^	159.68± 3.04^aa^ ^,^ ^cc^ ^,^ ^dd^			
D	161.85± 10.24	292.85± 21.01^bb^	239.28± 27.64^a^ ^,^ ^bbc^	193.19± 23.81^a^ ^,^ ^cc^	176.36± 15.70^cc^ ^,^ ^d^			
E	169.48± 7.09	280.22± 33.07^bb^	190.51± 18.89^aa^ ^,^ ^cc^	192.10± 24.04^a^ ^,^ ^c^	177.00± 9.39^cc^			
F	168.49± 15.77	285.08± 20.93^bb^	308.26± 30.87^bb^	238.36± 24.45^bb^ ^,^ ^d^	192.75± 10.46^cc^ ^,^ ^dd^			
P						0.000	0.000	0.000

Shapiro–Wilk tests confirmed that the IL-6 data across all groups followed a normal distribution. The results of the two-way repeated-measures ANOVA are presented in Table 7. A significant interaction effect between time and group was observed (p < 0.01, partial η2 = 0.472), along with significant main effects of both time (p < 0.01, partial η2 = 0.854) and group (p < 0.01, partial η2 = 0.733).

Superscript annotations ^a^: *p* < 0.05 vs. control group at the same time point.

aa : *p* < 0.01 vs. control group at the same time point.

b : *p* < 0.05 vs. pre-exercise within the same group.

bb : *p* < 0.01 vs. pre-exercise within the same group.

c : *p* < 0.05 vs. immediate post-exercise within the same group.

cc : *p* < 0.01 vs. immediate post-exercise within the same group.

d : *p* < 0.05 vs. 24h post-exercise within the same group.

dd : *p* < 0.01 vs. 24h post-exercise within the same group.

Post hoc multiple comparisons indicated that the CWI group demonstrated the most effective suppression of IL-6 levels, followed by the FES group, with a statistically significant improvement observed at 48 h post-exercise (p = 0.000, p < 0.01, 95%CI:−123.52, −55.50).

#### 3.2.3 CK

Shapiro–Wilk tests confirmed that the CK data in all groups followed a normal distribution. Results from the two-way repeated-measures ANOVA are summarized in Table 8. The interaction effect between time and group was not significant (p > 0.05, partial η^2^ = 0.242). However, there was a significant main effect of time (p < 0.01, partial η^2^ = 0.780) and a significant main effect of group (p < 0.05, partial η^2^ = 0.545).

**TABLE 8:** Changes in CK concentration across groups and time points (mean ± SD, U/L).

Group	Pre-exercise	Post-instant	Post-24 h	Post-48 h	Post-72 h	Time effect:F	Group effect:F	Interaction effect:F
A	143.31±15.01	245.73±35.18^b^	192.73±22.87^a^	168.83±15.94^a^ ^,^ ^b^	132.06±11.01^a^ ^,^ ^b^	84.907	5.750	1.536
B	139.48±16.77	305.85±51.98^bb^	231.71±30.20^bb^	189.22±8.29^a^ ^,^ ^bb^ ^,^ ^c^	154.53±30.91^cc^ ^,^ ^d^			
C	147.94±13.62	248.24±55.30^b^	201.18±19.94^a^	182.96±13.49^a^ ^,^ ^b^	145.53±16.32^c^			
D	138.75±12.31	278.51±53.72^b^	227.04±27.36^b^	184.47±18.48^a^ ^,^ ^b^ ^,^ ^c^	159.59±15.35^c^ ^,^ ^d^			
E	149.33±14.41	329.16±78.02^bb^	223.20±34.09^b^ ^,^ ^c^	177.02±11.74^a^ ^,^ ^cc^	148.45±18.91^cc^ ^,^ ^d^			
F	137.95±14.03	274.04±65.70^b^	276.88±66.05^bb^	231.18±30.22^bb^	181.22±10.78^b^ ^,^ ^c^ ^,^ ^d^			
P						0.000	0.001	0.150

Superscript annotations ^a^: *p* < 0.05 vs. control group at the same time point.

aa : *p* < 0.01 vs. control group at the same time point.

b : *p* < 0.05 vs. pre-exercise within the same group.

bb : *p* < 0.01 vs. pre-exercise within the same group.

c : *p* < 0.05 vs. immediate post-exercise within the same group.

cc : *p* < 0.01 vs. immediate post-exercise within the same group.

d : *p* < 0.05 vs. 24h post-exercise within the same group.

dd : *p* < 0.01 vs. 24h post-exercise within the same group.

Post hoc multiple comparisons indicated that the massage group exhibited the most effective reduction in CK levels, suggesting superior recovery from exercise-induced muscle damage, with a statistically significant improvement observed at 48 h post-exercise (p = 0.000, p < 0.01, 95%CI:−97.58,−27.12).

## 4 Discussion

### 4.1 Overview

**FIGURE 3:**
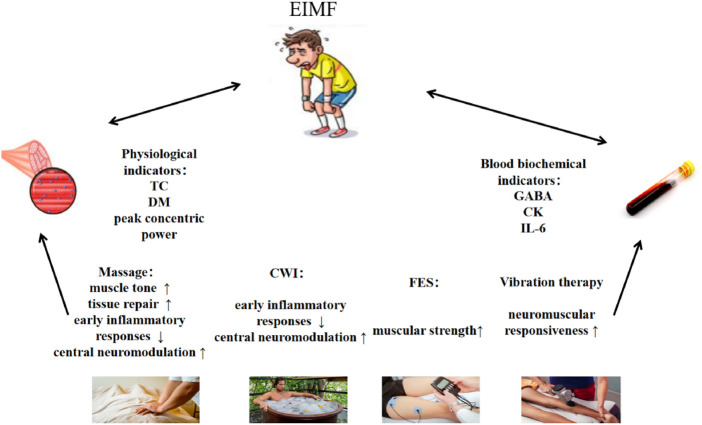
Mechanistic pathways and intervention strategies for exercise-induced muscle fatigue.

This study aimed to compare the effectiveness of massage, CWI, FES,and vibration therapy in alleviating exercise-induced muscle fatigue, using physiological and biochemical indicators. As shown in Figure 3, the results showed that vibration therapy had a significant effect on improving muscle responsiveness; massage effectively improved muscle tone; FES demonstrated the best performance in enhancing muscle strength; both massage and CWI showed advantages in suppressing early inflammatory responses and central nervous system modulation; and massage therapy was also found to significantly promote tissue repair.

### 4.2 Effects of different recovery interventions on physiological responses related to exercise-induced muscle fatigue

#### 4.2.1 TC

TC refers to the duration from the onset of a neural signal to the completion of muscle contraction. It is a key indicator of neuromuscular conduction efficiency and muscle response speed (Martín-Rodríguez et al., 2017).

Vibration therapy effectively reduced TC at 72 h post-exercise, indicating an improvement in neuromuscular responsiveness. This effect may be attributed to the promotion of capillary dilation, increased local blood flow and oxygen delivery, and enhanced lymphatic circulation (Park et al., 2015), which accelerate the clearance of lactate and other metabolic waste products, thus facilitating muscle recovery (Kang et al., 2017). Additionally, a meta-analysis suggested that vibration therapy can stimulate the central nervous system (Borzuola et al., 2023), enhancing motor unit recruitment efficiency and shortening contraction time. This adaptation may be related to an increased cross-bridge formation rate and faster release of troponin, allowing muscles to reach peak contraction more quickly (Chwała et al., 2021).

Except for vibration therapy, the massage group showed near-complete recovery of TC at 48 h post-exercise. This effect is likely due to the enhancement of venous return through massage, which reduces lactate accumulation and improves blood flow and oxygen delivery, allowing muscle fibers to respond more effectively to neural stimuli, thus decreasing TC. Additionally, massage can reduce passive muscle tension, alleviate muscle cramps (Dakić et al., 2023), and improve motor unit coordination. These findings are also supported by a meta-analysis, which demonstrated that post-exercise massage relaxation can effectively enhance muscle recovery and performance.

#### 4.2.2 DM

DM reflects the greatest displacement of muscle fibers during contraction and serves as an important indicator of muscle flexibility and elasticity (Čular et al., 2023). Immediately following intense physical exertion, increased muscle tension and stiffness due to forceful contractions often lead to a marked reduction in DM values.

Although no significant differences were observed in DM due to its smaller values, we observed that the massage group nearly fully recovered to baseline DM levels at 48 h post-exercise, whereas other intervention groups had not reached comparable recovery levels. This finding is consistent with a meta-analysis by Guo et al. (2017) (Guo et al., 2017)which demonstrated that massage effectively alleviates subjective muscle soreness closely associated with muscle stiffness. The mechanical effects of massage may help reduce intramuscular fluid (edema) and release fascial restrictions, thus improving muscle elasticity and facilitating the recovery of DM values.

#### 4.2.3 Peak concentric power

Peak concentric power refers to the maximal force output during concentric muscle contractions and serves as an important indicator of muscle strength and explosive power recovery. Immediately post-exercise, muscle strength typically declines significantly due to fatigue. At 24 h post-exercise, this reduction is often exacerbated by ongoing acute metabolic disturbances and inflammatory responses.

This result is consistent with previous studies, which have shown that FES can counteract the functional decline caused by exercise-induced muscle fatigue by enhancing muscle contraction capacity and increasing motor unit recruitment efficiency. The primary mechanism involves FES-induced activation of alpha motor neurons (Enoka et al., 2020)、increased excitability of fast-twitch muscle fibers (Smith et al., 2003), reduced muscle viscosity, and improved muscle elasticity (Rahmati et al., 2021). Furthermore, the electrical pulses from FES can improve local blood circulation, promote energy metabolism, accelerate the resynthesis of adenosine triphosphate (ATP) and phosphocreatine (PCr), and enhance calcium ion release and actin-myosin cross-bridge formation [ (Sañudo et al., 2020). These mechanisms collectively contribute to increased muscle contraction efficiency, significantly enhancing maximal strength and explosive power (Wiltshire et al., 2010; Barnett, 2006; Pinar et al., 2012).

### 4.3 Effects of different recovery interventions on biochemical markers related to exercise-induced muscle fatigue

#### 4.3.1 GABA

GABA acts as the primary inhibitory neurotransmitter in the central nervous system, responsible for balancing neuronal excitability. Immediately post-exercise, the nervous system is in a heightened state of excitation, and the body increases GABA release to reduce excessive activation and subsequently lower neuronal excitability.

This study found that massage therapy had a significant effect on the recovery of GABA levels, followed by CWI. Massage exerts mechanical pressure on muscles and fascia, reducing the excitability of motor neurons, stimulating the vagus nerve, enhancing GABA synthesis, and inhibiting the release of stress-related hormones such as cortisol (Diego et al., 2004), promoting relaxation (Field, 2014). Additionally, massage can improve local blood circulation, reduce the release of pro-inflammatory cytokines such as IL-6, accelerate the clearance of lactate and inflammatory mediators, thereby alleviating neuroinflammation and indirectly enhancing GABA’s inhibitory effects.

In addition to massage, we also observed that CWI effectively reduced neuronal excitability within 24 h post-exercise. The mechanism of action is thought to involve a reduction in muscle and nerve temperature through CWI (Vaile et al., 2011), slowing synaptic transmission, inhibiting excessive neuronal activation, and activating the parasympathetic nervous system (López-Ojeda and Hurley, 2024), This leads to decreased cortisol secretion (Algafly and George, 2007), This leads to decreased cortisol secretion (Peake et al., 2017), reduces anxiety and muscle tension (Shevchuk, 2008), and indirectly facilitates GABA recovery.

#### 4.3.2 IL-6

During exercise, skeletal muscles synthesize and release large amounts of IL-6 into the bloodstream. IL-6 exhibits biphasic immune-modulatory effects, acting as a pro-inflammatory cytokine in the early stages and as an anti-inflammatory mediator in the later stages. Initially, IL-6 works synergistically with tumor necrosis factor-alpha (TNF-α) and interleukin-1β (IL-1β) to trigger acute inflammation and promote muscle repair signaling. Subsequently, IL-6 induces the secretion of anti-inflammatory cytokines such as IL-1 receptor antagonist (IL-1ra) and IL-10, while inhibiting the activity of TNF-α. This rapid transition helps prevent excessive inflammation and promotes a systemic low-grade anti-inflammatory environment, which is a hallmark of immune adaptation to exercise-induced stress (Ten Broek et al., 2010; Nash et al., 2023).

In this study, CWI demonstrated the most significant effect in reducing IL-6 levels, followed by FES. This finding is consistent with a meta-analysis by Olivier Dupuy et al. (Dupuy et al., 2018), which confirmed that CWI effectively attenuates post-exercise inflammatory responses. The primary mechanism is thought to involve cold-induced vasoconstriction, which reduces the overexpression of IL-6 and the release of other inflammatory cytokines, thereby limiting immune cell infiltration and effectively suppressing acute inflammation (White and Wells, 2013). However, our data also suggest that the reduction in blood flow due to cold exposure may impair sustained anti-inflammatory effects. At 48 h post-exercise, IL-6 levels in the CWI group were significantly lower than baseline, indicating rapid suppression of acute inflammation. An alternative explanation in studies on CWI is the hydrostatic pressure generated by the water, which may promote the clearance of inflammatory mediators from muscle tissue, synergizing with vasoconstriction. However, by 72 h, IL-6 levels rebounded to baseline, potentially reflecting a reduction in perfusion and the activation of endogenous regulatory feedback mechanisms (Bleakley and Davison, 2010). This may also represent a delayed initiation of the inflammation-mediated regeneration signaling phase, which could have been inhibited early on by CWI. This highlights a potential trade-off between suppressing acute inflammation and potentially interfering with long-term adaptive signaling pathways. Therefore, prolonged and strong suppression by CWI may act as a double-edged sword, reducing acute inflammation at the cost of disrupting long-term adaptive signaling necessary for muscle repair and growth.

In addition to CWI, FES also significantly reduced IL-6 concentrations within 24 h post-exercise. The potential mechanism may involve activation of the sympathetic nervous system and the initiation of anti-inflammatory signaling pathways (Do Carmo Almeida et al., 2018), which decrease the release of pro-inflammatory cytokines and limit secondary inflammation associated with exercise-induced muscle microdamage.

#### 4.3.3 CK

CK is a widely recognized biochemical marker for assessing muscle damage. Following intense physical activity, disruption of the muscle fiber cell membrane leads to a substantial release of CK into the bloodstream. Elevated CK levels are indicative of the severity of muscle cell damage (Brancaccio et al., 2007).

This study found that massage had a significant effect in promoting the reduction of CK levels. This result not only addresses the limitation of previous studies, which had short observation periods focusing mainly on the first 24 h, but also further supports the key role of massage therapy in reducing muscle damage and promoting tissue repair. Based on relevant research, we summarize three main mechanisms by which massage promotes CK recovery: First, massage promotes blood circulation, alleviates muscle tension, and enhances lymphatic flow, which accelerates cell repair, promotes CK metabolism, and facilitates renal excretion. Second, massage reduces microscopic muscle damage and associated inflammation by decreasing muscle membrane permeability, thus limiting further leakage of CK into the bloodstream (Brancaccio et al., 2007). Third, massage has been shown to regulate the expression of pro-inflammatory cytokines, helping to reduce local inflammation and enhance tissue regeneration. Together, these mechanisms contribute to minimizing CK and inflammatory mediator retention in the damaged tissue microenvironment (Weerapong et al., 2005), thereby facilitating a more efficient and rapid recovery process. This finding is consistent with conclusions from multiple meta-analyses. For example, a meta-analysis by Guo et al. (2017) concluded that massage effectively alleviates delayed-onset muscle soreness and reduces CK levels following intense exercise. Similarly, Poppendieck et al. (2016) (Poppendieck et al., 2016) reported a moderate effect of massage on muscle performance recovery. Our results provide further experimental support for these large-scale evidence-based conclusions and reinforce the clinical application value of massage in muscle recovery.

### 4.4 Limitations

This study has several limitations that should be acknowledged. First, the primary limitation is the small sample size in each group (n = 5). Although *a priori* power analysis conducted with G*Power indicated that a total sample of 30 participants would provide adequate power to detect the overall group × time effects within a repeated-measures framework, the small group size substantially reduced the statistical power of *post hoc* pairwise comparisons. This limitation was further compounded by the application of Bonferroni corrections and the restricted degrees of freedom, leading to wider confidence intervals and an increased risk of Type II errors (false negatives). Therefore, the present findings should be considered preliminary and require confirmation in larger cohorts.

Second, our study included only healthy young adult males to control for confounding variables such as sex hormones and age-related neuromuscular changes. While this homogeneity enhances internal validity, it limits the generalizability of the results to female athletes, older adults, or different athletic populations.

Third, the duration of each recovery intervention was not standardized, as they were based on best practice guidelines for each specific modality. This presents a potential confounding variable, as we were unable to fully separate the effects of intervention type from its duration. Future studies could explore the dose-response relationship for each modality.

Finally, the follow-up period was limited to 72 h, capturing the acute recovery phase but lacking information on the long-term effects of these strategies on training adaptation.

## 5 Conclusion

The results of this study showed that massage provided the most comprehensive recovery benefits, including central nervous modulation (GABA), improved neuronal excitability, accelerated CK clearance, and reduced muscle tension. CWI was particularly effective in inhibiting excessive IL-6 secretion and rapidly controlling acute inflammation, although its effects fluctuated in the later stages of recovery. FES significantly enhanced muscle explosive power and peak concentric performance. Vibration therapy demonstrated a clear advantage in shortening TC, indicating enhanced neuromuscular responsiveness. The static stretching group did not show significant benefits. This may be because static stretching primarily enhances range of motion (ROM) and flexibility, while providing limited facilitation of metabolite clearance and neuromuscular activation. Moreover, under typical stretching conditions, acute static stretching can induce transient reductions in force production, thereby diminishing its effectiveness for strength and power recovery.

This study provides novel evidence highlighting the differential regulatory effects of various recovery interventions across the central-peripheral-immune dimensions of post-exercise fatigue. These findings advance the mechanistic understanding of recovery and regeneration strategies. Based on the type of exercise-induced damage and specific recovery objectives, practitioners should adopt a targeted or combined intervention approach to optimize recovery outcomes.

## Data Availability

The raw data supporting the conclusions of this article will be made available by the authors, without undue reservation.
